# A History of the Alexithymia Concept and Its Explanatory Models: An Epistemological Perspective

**DOI:** 10.3389/fpsyt.2019.01026

**Published:** 2020-01-31

**Authors:** Francisco López-Muñoz, Francisco Pérez-Fernández

**Affiliations:** ^1^ Department of Psychology, Faculty of Education and Health, University Camilo José Cela, Madrid, Spain; ^2^ Neuropsychopharmacology Unit, Hospital Doce de Octubre Research Institute (i+12), Madrid, Spain; ^3^ Portucalense Institute of Neuropsychology and Cognitive and Behavioural Neurosciences (INPP), Portucalense University, Porto, Portugal; ^4^ Thematic Network for Cooperative Health Research (RETICS), Addictive Disorders Network, Health Institute Carlos III, MICINN and FEDER, Madrid, Spain

**Keywords:** alexithymia, history, therapeutic models, psychosomatic medicine, theoretical constructs

## Abstract

Alexithymia, as a theoretical psychotherapeutic construct, finds its origins in psychosomatic medicine, actually being quite old. However, beyond the specific observations and case studies, their characterization and systematization is relatively recent. However, from an epistemological point of view, it remains the subject of debate and therefore remains outside the conventional diagnostic guidelines. Possibly, its history, closely linked to psychoanalysis, as well as the lack of clear empirical references, has turned the alexithymia construct before into a good descriptive and comprehensive framework than in a precise diagnostic model. In this article it is, following the thread conduits of the historical perspective, to deepen these epistemological aspects.

## Introduction

Psychosomatic medicine in general is based on the principle that emotions and personality have an impact on bodily functions, and thereby play a part in physical wellbeing or illness. Nevertheless, because the mechanisms of these interactions are not clear, very few of its explanatory models have gained the consideration of research paradigm (1). In fact, today’s psychosomatic model dates back to the explanations provided by Sigmund Freud (1856–1939) on the understanding of neurosis, whereby it has its origin in the theoretical bases of the orthodox psychoanalytical model that summarily contends that the intrapsychic conflict that explains both the genesis and development of the structuring of personality, together with its pathologies, prompts a high level of emotional activation that may cause more or less serious damage to the organism (2). This hypothesis has been widely debated for decades inasmuch as it was complicated to establish clear empirical references to support it, which gave rise to far-reaching controversies that even affected the very development of psychoanalysis as a school, giving rise to a broad diversification of the heterodoxy (3). Nevertheless, the appearance of the construct of alexithymia appeared to go some way to resolving many of these inconsistencies by finding quantifiable empirical referents that rekindled the interest in different biological aspects of Freud’s controversial theory (4).

Nonetheless, and beyond Freud, the issue enjoys a long-standing medical tradition, which would in due course be consolidated within the field of psychiatry, as throughout the 19^th^ century it seemed clear that emotion, personality, and health were closely related. This meant that professionals soon raised the question of how to establish a two-way nexus between mental functions and physical states (5). Thus, for example, the US surgeon William Beaumont (1785–1853) established that fear and anger reduced the amount of gastric mucus secreted (6). The same may be said in relation to the first controlled studies on hypnosis, as a British surgeon called James Braid (1795–1860) used experimental means to prove that patients subject to hypnotic suggestion suffered less physical pain during surgical procedures (7). It therefore stands to reason that a school of medical inspiration in origin, such as Freudian psychoanalysis, and inspired by the work undertaken by the neurologist Jean-Martin Charcot (1825–1893) in La Salpêtrière Hospital in Paris, should embrace these and other findings in its explanation of the causes and consequences of neurotic processes.

The widespread dissemination of approaches of this nature, which converged in the psychosomatic hypothesis, specifically took place in the 1940s and 1950s by the hand of doctors trained in psychoanalysis, such as Franz Gabriel Alexander (1891–1964) and Felix Deutsch (1884–1964), among others (1). Indeed, the psychosomatic paradigm added emphasis to the role of conflicts of the unconscious by generating states of chronic activation in patients in physiological pathologies, and even in some kind of tissue alterations. As is logical, this hypothesis, focusing on the organic effects of emotion, involved a new approach to therapy, inasmuch as it was assumed that together with psychogenic and biological aspects, environmental stresses would also have an important part to play in the origin and development of all nature of complaints, as factors that would also alter an individual’s emotional state to a greater or lesser extent (8). We should note, however, that despite the importance these authors gave to the intrapsychic conflict, the aim here was not to uphold a psychoanalytical approach linked to Freudian orthodoxy, as the pathogenic mechanism would not be actually symbolic, but instead of a psychobiological nature (9).

Whatever the case, the psychosomatic model based on an individual’s internal conflict was backed by scant empirical evidence, whereby it began to be seriously questioned toward the end of the 1950s; above all because the therapeutic model based on psychoanalytical models did not appear to provide results that were both uniform and consistent. Although certain patients made headway in reducing the physical symptoms of their disorders, others, especially when they manifested development-related problems, only appeared to record clear benefits related to their physiological problems when they underwent other kinds of psychodynamic therapies of psychological support, such as the well-known brief psychodynamic therapy. In fact, some even worsened when they were prompted to look for the unconscious reasons for their motivations, a situation that ultimately raised doubts about the psychosomatic approach itself (10, 11).

## Alexithymia as a Model

The crisis of the therapeutic model based on psychoanalysis for addressing psychosomatic problems did not question the existence of such problems, as their clinical evidence seemed clear and consistent, but instead the way of explaining them with a view to seeking alternative approaches. This means that the concept of alexithymia was not the result of an eureka moment, but instead emerged in the early 1970s following a protracted period of reflection and the evaluation of sundry alternative hypotheses (1).

Back in 1948, a psychiatrist of Swiss origin called Jürgen Ruesch (1910–1995) reported finding a unique casuistic among his patients: those that experienced topical psychosomatic complaints—such as migraines, abdominal disorders, and hypertension, as well as other chronic physiological complaints, were characterized by being scarcely imaginative, as well as having problems with the verbal and symbolic expression of emotions different to those usually found in patients with disorders other than somatic symptoms (12). Before being seen as a disorder of neurotic origin, this problem was considered a difficulty arising from a deficient personality development, understanding this to be the main cause of psychosomatic disorders. Along these same lines, and around the same time, authors such as the US neuroscientist Paul MacLean (1913–2007), known for his popular triune brain theory, contended that patients with psychosomatic complaints encountered difficulties in expressing their feelings, speculating with the possibility that this might be due to a weak consolidation of the areas of the neocortex related to language processing: the emotions that caused the patient significant distress were not suitably symbolized, whereby they were manifested through what was referred to as “organic language” (13).

In the early 1950s, even psychoanalysts themselves began to encounter a growing number of cases that broke with the traditional model of intrapsychic conflict. Thus, for example, Karen Horney (1885–1952) first reported that some of her patients were immune to psychoanalytical treatment due to a significant lack of emotional awareness, little expression of their internal states, a minimal interest in their dreams and fantasies, excessive focus on thoughts and, as a result, pursued a superficial life-style focused on outside experience (14). Nonetheless, what was of interest to Horney was that this type of patient was prone to develop psychosomatic problems, eating disorders, polydrug use and, in general, all kinds of compulsive behaviors that, it seems, were closely related to the systematic avoidance of the understanding of their own emotions and a profound feeling of emptiness. What is important, at this point, is that despite admitting that these people did not fit into the traditional model for explaining neurosis, nor respond well to psychoanalytical therapy, Horney sought to attribute their behavior to the existence, in these individuals, of powerful and deeply rooted defenses against unconscious conflicts (1). This approach began to change among the advocates of psychoanalysis barely a decade later, with the appearance of specialists that began to reject the model of neurotic defense in favor of a more eclectic approach. This was when the notion began to be accepted that these patients’ behavior would involve, above all, personality deficits: given the lack of a rich inner life, these people focused their concerns on their organic symptoms and the medium’s influence, thus generating a model of “operational thinking”, which ended up having a very negative impact on their interpersonal skills (15, 16).

Nevertheless, and despite the increasing amount of data on this specific type of patient in the clinical literature, it was not until the mid-1960s that the US psychiatrist John Case Nemiah (1918–2009) and his Greek-born colleague Peter Emanuel Sifneos (1920–2008) jointly embarked upon a systematic study of the cognitive style of a sample of individuals with common and persistent psychosomatic problems (17, 18). They came to the conclusion that compared to individuals with standard mental disorders, many of those that reported these associated physiological complaints found it extremely difficult to describe their subjective feelings, apart from having an impoverished fantasy, as well as a utilitarian cognitive style focusing on the outside. This unusual psychological concept was what in 1972 Sifneos coined as ‘alexithymia’, which is formed by the roots of several Greek words, and literally means “lack of words for emotion” (18–20).^1^

Following the publication of these initial empirical findings, alexithymia began to quickly gain in popularity among practitioners of psychiatry and psychology. This was especially the case after two international conferences; the first held in London in 1972 and the second in Heidelberg in 1976, which established the importance of the alexithymia model in research into states of emotional deficit (18). In fact, following the London conference, it became clear that despite the contradictory views held by clinical and non-clinical specialists, there was a need to give the term uniformity when addressing problems of this kind if the aim was to ultimately find a framework of consensus that would pave the way for communication between basic and applied science. Hence it was agreed to replace the fuzzy concept of ‘affection’ by the more precise term of ‘emotion’ for all its somatic aspects, while the term ‘feeling’ was reserved for its cognitive components (18).

The truth is that a clear convergence swiftly appeared in the literature between a vast number of therapists and the model propounded by Nemiah and Sifneos. But it was not alone. The result was that the first empirical studies on alexithymia provided unidirectional results that soon consolidated a clear explanatory model based on four elementary premises: 1) difficulty in identifying and describing feelings; 2) difficulty in distinguishing between feelings and bodily sensations related to emotional activation; 3) restrained and limited imaginative processes, adopting the guise of an impoverished fantasy; and 4) a cognitive style oriented toward the outside (1). It is worth stressing, nonetheless, that alexithymia seemed to manifest itself in an unusual and paradoxical manner, as at first glance the patients appeared to express a strong sense of melancholy, with chronic dysphoria, or else outbursts of tears and anger. Nevertheless, following an in-depth interview, a specialist found that in reality these people knew quite little about their internal states and were often incapable of effectively relating them to memories, specific situations, or recurring fantasies (9). The important thing is that the approach taken by Nemiah and Sifneos allowed superseding the classical psychoanalytical proposal that used the link between the repressed effect and the somatic symptoms to shift the understanding of alexithymia toward a view close to developmental psychology: an alexithymic one, due to an inefficient psychological development that converged in a dysfunctional adult personality, would experience serious difficulties when cognitively integrating the effects, which would stop the patient being properly regulated and modulated. This would prompt, on the one hand, the usual communicative problems that are typical of the disorder and, and on the other, a clear vulnerability to experience a growing tension informed by non-differentiated states of unpleasant emotional activation (22).

Indeed, certain cases of alexithymia provided evidence of neurotic conflict in the classical sense of the concept, but it was soon evident that alexithymia could both be found in the very origin of these conflicts and strengthen others arising during its development, thereby revealing that the main problem of alexithymia was not so much one of neurosis, but the poor emotional regulation that its emotional deficits caused and, furthermore, pointed to an organic substrate (23). In view of this state of affairs, it was perfectly possible that intrapsychic conflicts could be one of the causes of the disorder, although they were undoubtedly not the main one, as psychoanalysts had believed for decades. In fact, alexithymia had transformed, largely, into describing someone with problems when cognitively processing emotions with complete independence of their origin and intensity. In fact, subsequent research both from the field of developmental psychology and from neuropsychology has found that the root of alexithymia could lie in either a problem of cerebral organization, or in a marked lack of suitable emotional models during the passage from childhood to adulthood, or else a combination of both factors (24, 25).

## A Debated Model

In view of the approaches considered, Sifneos soon proposed a general etiological model for alexithymia that, to begin with, subdivided it into two types: primary and secondary. The primary one had its origin in a neuroanatomical or physiological defect, possibly hereditary in nature, whereby the communication between the limbic system and the neocortex would not function properly, affecting hemispherical lateralization, which would lead to an inability to associate fantasies, thoughts, and languages with emotions. The secondary one, in turn, is associated with psychological traumas in childhood, serious and prolonged traumatic aggressions in adulthood, or psychodynamic sociocultural factors that prompt an overuse of defensive mechanisms such as repression or negation (19, 21). Whatever the case, and without a clear empirical corroboration, this etiological explanation was based solely on clinical observations and case studies. Hence the reason that, in the first instances, the model of alexithymia proposed by Nemiah and Sifneos faced major opposition.

The first thing that was challenged was the fact that the proposal was based, as we have already stated, on scant empirical information, which meant there was no point in validating it inasmuch as it had not been properly verified (1). Indeed, it was a valid criticism insofar that many clinicians believed they had found an explanatory panacea for the somatic symptoms, which led to the misinterpretation of their own particular clinical experiences as a corroboration of the construct’s validity when, in reality, what was happening was that they appeared to be fitting them subjectively to a pre-established model that had never been verified (26). Yet the second great reticence caused by the model of alexithymia was that it led to the commission of a serious mistake in interpretation: many researchers assumed the existence of a specific and consolidated relationship between alexithymia and the whole known raft of classic psychosomatic symptoms, when the truth is that the construct had been defined, rather than as a cause, as a factor of risk. Alexithymia thus heightened the patient’s vulnerability toward psychosomatic disorders, but the efficient variables that prompted were others linked to the management of emotions, such as badly resolved situations of grief, poverty, and abandon, for example (2). In view of this, the most critical views estimated that the so-called “alexithymic characteristics” could be explained more effectively through directly observable factors, such as socioeconomic status or other variables of a situational nature, such as adverse life events, which prompted the patient to adopt systemized defensive strategies of resistance, negotiation, and negation, which again opened the door to a psychodynamic interpretation of the problem (27). This is the context that gave rise to new and popular psychoanalytical reviews of the construct, such as the one proposed by the New Zealander Joyce McDougall (1920–2011), who coined the phrase “emotionally deaf and dumb” to refer to patients suffering from alexithymia. McDougall, who chose to elude the intrapsychic conflict to concentrate on the problem of symbolic linguistic representation, linked alexithymia, and psychosis when finding that a psychotic and alexithymic patient treated language in a similar, but opposite way, which possibly means that both their pathological manifestations had a common origin. She thus explained that the psychotic patient tries to make up for their psycho-emotional deficits through the delirious use of words in order to overcome their anxiety, while the alexithymic patient tackled their anxiety by emptying their words of emotional meanings, which induced the psychosomatic disorder. This explained, in her view, why the psychotic patient expressed their delirium in a mental order, while the alexithymic one expressed their delirium in a physiological way (28).

On the other hand, and as an alternative critical pathway, transcultural studies had established the notion that neither the psychosomatic symptoms nor the verbal expression of emotions were universal, being mediated by specific cultural attitudes toward life events, as well as by the restrictions imposed by language in each case. Nevertheless, the response to this objection by the advocates of the model of alexithymia was that it was unlikely that sociocultural and language aspects could explain manifestations of alexithymia such as the reduction in imagination, impoverished fantasy, or poor symbolization, and they reproached their critics, who were generally behavioral therapists, that their analysis model did not even consider these kinds of hypotheses insofar as it had either clearly ignored them or studied them only superficially and with little theoretical rigor (1). Accordingly, those that defended the construct would say that they were not in this case faced with a question of “believing or not believing” in it, or of a problem intrinsically related to its internal validity, but instead with an epistemological difficulty that was due rather to the type of scientific paradigm it was analyzed from (29). In fact, other dimensional models based on internal hypotheses had proven their utility within the clinical field and in psychopathological research such as, for example, that of introversion-extroversion, and this was due to the fact that, like alexithymia, they should not be understood as a mere diagnosis, but instead as a personality trait (1). Nevertheless, Sifneos was convinced of the construct’s validity, and of the fact that sooner or later he would find empirical proof of the same, as in 1973 he introduced a simple questionnaire, the *Sifneos Alexithymia Questionnaire* (SAQ) to be administered to patients in daily clinical practice. This screening test consisted of 17 items, of which eight referred to key issues for revealing a patient’s symptoms of alexithymia (30). This instrument was subsequently reformulated by Sifneos himself, converting it into the *Beth Israel Hospital Questionnaire* (BIQ-1), comprising 21 items to be completed by the actual therapist based on their observations, which raised doubts about its objectivity insofar as the possibility of obtaining emotional results from the patient varied depending on the interviewer’s experience (20, 31).

Indeed, neuropsychological research came to the aid of the alexithymia paradigm inasmuch as it detected, for example, that those patients that underwent a commissurotomy, or surgical incision into the callosal commissure, experienced, as the model predicted, many of the cluster of symptoms traditionally associated with the complaint: the visual-spatial information, with a major emotive content, processed by the right hemisphere of the brain, no longer travelled freely to the left hemisphere to be sequentially segmented and reformulated in a verbal format. These patients therefore encountered all kinds of difficulties when symbolizing, imagining, fantasizing, or expressing themselves emotionally, which made alexithymia, at least in functional terms, into a phenomenon that was the complete opposite to creativity (32).

In sum, the verbalization and use of emotions involved a transfer of data, with a transduction or change in the type of information contained in the same, between the limbic system and the cortex, as well as between both hemispheres of the brain, which is precisely the problem affecting the alexithymic patient. Consequently, the progressive clarification of the brain structures involved in the processing of emotional activation shed empirical light on the model, at least in part, and helped to explain the fact that alexithymia often appeared in patients linked to substance abuse, sociopathic or borderline personality traits, eating disorders, panic attacks, somatoform disorders, or psychogenic pain (18). Moreover: as opposed to Horney’s aforementioned opinion, attributing the shortcomings of psychoanalysis in these individuals to a firmly entrenched resistance to the unconscious, what really explained why traditional psychodynamic therapies fell short with these patients, specifically those of a primary nature, was that because of their organic difficulties for imagining, fantasizing, or visualizing situations, such therapeutic approaches were of little use and could even be counterproductive.

## The Consolidation of the Construct

The advancement in the understanding of the neurological bases of alexithymic manifestations, as well as the slow but steady progress made in the clinical field, prompted numerous attempts to strengthen it through theoretical refinement and the collation of data gathered from exploratory sample studies. The first attempt, barely concealed among many professionals, involved dissociating insofar as possible alexithymia from the temptation to explain it solely in psychoanalytical terms and, of course, reinforce it in such fields as neuropsychology, developmental psychology, and personality psychology. This gave rise, to mention just one example, to studies that sought to influence the emotional symbolic development of children and adolescents, as well as their expression of emotions, in connection with the stages of development described by Jean Piaget (1896–1980). As is common knowledge, Piaget contended that psychological growth began with the acknowledgement of one’s own physiological bodily conditions, and culminated in the cognitive recognition of the psychological states of others, whereby psycho-emotional development and the communication of emotions were key elements both in individuals’ cognitive development and in the proper formation and consolidation of their personality (33). In short, the well-consolidated higher psychological processes that are the hallmark of the healthy adult, as propounded early on by the Austrian doctor Max Schur (1897–1969), were only possible largely through a progressive desomatization of the individual (34).

Based on these approaches, of psychoanalytical resonance in origin, subsequent developmental psychology focused its attention on, among other things, the way of symbolizing and integrating emotions, as well as their communicative functions, in keeping with the cognitive mechanisms involved in their regulation and modulation (22). This involved a basic line of research for the theoretical consolidation of the model of alexithymia inasmuch as, it seems, these mechanisms were absent, or were at least dysfunctional, in alexithymic patients, which rendered them vulnerable to the stresses generated in the non-differentiated states of emotional excitement that explained the disorder (1). Nevertheless, and in view of its own diagnostic conditions, given that it involved a descriptive concept based not on an illness but instead informed by the patient’s complaint of one or more basic pains, there persists—and still persists in some way—the handicap of deciding whether alexithymia could be considered a personality trait in the true sense of the meaning or a state, which prompted the need to suitably evaluate both aspects (35). The fact that alexithymia was linked to different psychosomatic complaints may have led to its premature acceptance, and not necessarily the right one, of the existence of a direct relationship between alexithymia and psychosomatic illness, which constituted a problem when assessing the construct because the clinical observation found both psychosomatic patients that were not alexithymic and alexithymic patients that did not record any psychosomatic malaise whatsoever (31). In fact, a constant when addressing therapy with these kinds of patients was the scant literature on their treatment, as individuals with alexithymia rarely sought help on their own accord, and generally ended up visiting a specialist at the behest of someone close to them who was frustrated by their communicative shortcomings or on the advice of a medical practitioner confused by their constant physical complaints that were difficult to determine and resisted traditional medical treatment (36).

This may explain why it was thought that the solution for addressing alexithymia, which in clinical practice was both a recurring and unquestionable phenomenon, would come from its controlled assessment through instruments. Sifneos himself, aware of the methodological limitations inherent to BIQ-1, did not take long to present BIQ-2, in this case a self-assessment scale consisting of semi-structured questions that patients were required to answer and, in theory, provided the therapist with first-hand information over and above that gathered during the clinical interview (37). This led to other measures such as the *Schalling-Sifneos Personality Scale*, the *Alexithymia Scale of Noël,* and the widely used *Toronto Alexithymia Scale* (TAS) (38). They were accompanied by structured interview procedures, such as the *Alexithymia Provoked Response Questionnaire* (APQR) (39), as well as the combined use of the appropriate tools with other common psychodiagnostic tests such as MMPI, TAT, and AT-9 in order to obtain more complete observations (31). All this led to a fairly accurate profile of an average person with alexithymia—generally a male who reported their first complaints at a mature age—as well as an epidemiological interest that revealed that this complaint was more frequent among the general population than initially thought, with the impact of alexithymia, furthermore, varying depending on the specific populations under study, but not in transcultural studies: more common among patients with psychosomatic complaints than among patients with other health issues; more common among those addicted to psychoactive substances and, in general, with an impact of between 8% and 10% in the normal undiagnosed population (31).

The gradual severance of the close bond that had historically been forged between alexithymia and psychosomatic pain allowed extending the problem’s horizon and, therefore, calibrating the construct’s validity in sundry fields, both psychosociological and cultural ones and regarding the field of health. Indeed, the focus began to turn toward alexithymia as a personal condition and not simply as a mere medical problem, or solely due to a diagnosable medical disorder. Thus for example, and given the shortcomings in the emotional management of alexithymia, it was thought that a parallel model of analysis could be established between alexithymia and psychopathy given the latter’s popular characterization by Hervey Cleckley (1903–1984), who among other things described a psychopath as having a significant deficit in the management and understanding of emotions. This led to a systemic study of the relationships between psychopathy and alexithymia in a sample of women by comparing the results obtained on the PCL-R psychopathy checklist of Robert Hare (b. 1934) and the measure of alexithymia provided by the TAS scale (40). Despite the similarity in the deficiency of the emotional manifestations affecting both constructs, and possibly constituting one of its main symptoms, it was found that they were not interchangeable insofar as they did not refer to the same thing, nor did they measure equivalent or correlative aspects of emotion.

The end result was a set of evidence that was difficult to doubt regarding the construct of alexithymia. The first and key piece of evidence was its undoubtable entity. In fact, although subject to permanent criticism, even its detractors had to accept its advantageous nature as an operational element, whereby a raft of clinical features subsumed beneath a single concept and systematically affected a specific group of disorders and patients with very specific characteristics (41). Thus, and despite its non-introduction—at least for the time being—in standard diagnostic classifications because of its debated status—trait versus state—those in favor of the model found a way of defending with assurances that although it could not be acknowledged as a diagnostic entity in its own right, it had the intrinsic value of characterizing and operationalizing a long history of psycho-medical observations of great social import which, until their appearance and systematization, had remained within the sphere of the undefined, thereby generating some considerable perplexity among specialists and the inconsistent treatment of patients (42).

## Recent Developments in Alexithymia

From the described perspective, recent research has focused on exploring the links of alexithymia, understood as a stable personality trait present in some patients, and various medical and psychiatric conditions. For example, a study of relationships between insight and alexithymia in adult outpatients diagnosed with obsessive-compulsive disorder (OCD) showed that the subjects of the sample that obtained higher scores on the TAS-20 scale also reflected poor or absent insight (43). Similarly, further work has established clear connections between body image disturbances and alexithymia in women with severe premenstrual dysphoric disorder (PDD) and patients with serious binge eating disorder (BDD). In the last case, moreover, the presence of alexithymia not only impoverished the evaluation and bodily satisfaction of individuals, but also seemed to increase depressive symptoms (44–46).

The relationships between alexithymia and other organic conditions have also been investigated, such as the presence of acute phase proteins—especially C-reactive protein— lipid levels, cholesterol, and cytokine imbalance, with special attention at the area of drug naïve outpatients diagnosed with other pathologies, such as OCD, major depression (MD), or panic disorder (PD). The results seem to establish links between alexithymic patients with OCD, MD, and PD and the presence of poor cholesterol regulation and high suicide ideation (47–49). Also, in line with the “stress-alexithymia hypothesis”, there seems to be a clear relationship between the high presence of C-reactive protein in blood and alexithymia. This connection motivates that the pro-inflammatory and anti-inflammatory cytokine balance may be tuned toward a pro-inflammatory imbalance with a concomitant altered cell-mediated immunity. These results point to the idea that the presence of alexithymic features in the patient should encourage more comprehensive therapeutic approaches, both medical and psychological, that could prevent the development of more severe diseases in alexithymic patients and, logically, improve their quality of life (50).

However, one of the main lines of research in the field is the risk of suicide, and there is a wide literature that seems to coincide in the fact that the presence of alexithymic traits in the individuals significantly increases suicidal behavior. Initially, it was thought that alexithymia, in suicides, would be associated mainly with depressive symptoms, so it could operate as a predictive factor for this type of behavior (51). Subsequent integrative studies have also shown that suicidal depressants with alexithymic traits—a fact that has also been found in relation to other psychiatric disorders as already indicated—showed elevated blood cholesterol levels, as well as deregulation of C-reactive protein and homocysteine. Consequently, this would confirm to some extent, and in the absence of further evidence, the predictions of “stress-alexithymia hypothesis” (52, 53): alexithymia could be a chronic condition of the patient, possibly due to childhood or early adolescence in that there were systematic abuses and/or abandonment, which would cause in the subject a state of pronounced inflammation with an impaired hypothalamic-pituitary-adrenal axis reactivity toward the usual stressors of daily life. Thus, the manifestation of alexithymia would operate as a chronic response to stress that would complicate both psychiatric disorders and other medical conditions (54).

## Conclusion: Brief Final Thoughts

To a certain extent, the historical debate on the issue of alexithymia is readily understandable when observed from an epistemological perspective, as it actually involves a rerun of the age-old conflict between basic and applied science, which in this case takes the form of a showdown between the therapist’s clinical observation and the critical eye of experimental methodology. Although it is true to say that Nemiah and Sifneos do not “discover” alexithymia—in fact, the latter has earned greater fame than the former by given it its name and investing more effort in its definition and disclosure— they should be granted the merit of managing to formulate a coherent explanatory model that integrates a long tradition of real and systematic medical observations that pointed toward a problem that basic research not only had failed to resolve, but had not even detected.

The collateral circumstance whereby the problem that Sifneos systemizes, more than psychological, constitutes a basically medical matter, had a great influence in its subsequent treatment when one considers the fact that, at least in its orthodox origin, psychoanalysis was the medical school of psychology par excellence. This was not only because it was inspired by a doctor such as Freud, but also because of its profoundly organic and deterministic approach incapable of understanding a mental problem that had not previously had a biological cause. Hence the reason that for a long time it attracted the interest of the general medical profession—and of psychiatry in particular—and also that the first treatments of these observations that would end up constituting alexithymia acquired a marked psychoanalytical and psychosomatic bias, closely interrelated, that would define its future. Possibly, the resistance that the psychopathology of a more empirical bias initially faced regarding the construct’s acceptance therefore involved a great deal of prejudice. It is possible that the issue in this case, more than its operative value, its empirical referents, or its epistemological underpinnings, lay in its debated and debatable origins.

The natural, and logical, tendency of basic research of not accepting models and approaches that do no not arise from within it led in this matter—as in others—to fierce discussions that, if we pay attention to the precedent, have less to do with the real therapeutic problem that alexithymia sought to define and operationalize than with its possible explanation and subsequent fit within the framework of the theoretical underpinnings of psychology and psychiatry. In fact, and this is very common among the more reductionist sectors of the psychological explanation, there is a tendency to forget with consummate ease that the psychological and the organic mutually influence each other, considering materialist explanations a single direction in this relationship, which tends to mean that they are often partial and confusing. It therefore involved, as is often the case with these confrontations that reignite the perennial conflict between science and practice, a debate on scientific primogeniture. Or, to put it another way, of a potentially sterile discussion between the nature of things and their value of use. Both questions may be perfectly legitimate, yet what is certain, as in the case that concerns us here, is that they are doomed to understand each other.

## Author Contributions

Both authors took part in planning the theoretical and conceptual basis for the study. FP-F wrote the first draft. Both authors took part in critically reviewing and editing the manuscript. 

## Conflict of Interest

The authors declare that the research was conducted in the absence of any commercial or financial relationships that could be construed as a potential conflict of interest.

## Footnotes

1
*α* (a) or “no”; *λέξις* (lexis) or “speech/word”; *θυμός* (zümós) or “emotion/mind”. While the noun *λέξις* in turn comes from the verb *λέγω*, which means “read”, which provides the double meaning (adapted from the Spanish version by Lusiardo and Rodrigo (21).
